# Clinical and sociodemographic determinants of disease progression in patients with nonalcoholic steatohepatitis in the United States

**DOI:** 10.1097/MD.0000000000028165

**Published:** 2021-12-17

**Authors:** Leonardo Ruiz-Casas, Gabriel Pedra, Anum Shaikh, Bethany Franks, Harpal Dhillon, João Diogo da Rocha Fernandes, Kamal Kant Mangla, Margarida Augusto, Jörn M. Schattenberg, Manuel Romero-Gómez

**Affiliations:** a HCD Economics, Daresbury, Cheshire, United Kingdom; b Novo Nordisk A/S, Søborg, Denmark; c Metabolic Liver Research Program, University Medical Center Mainz, Mainz, Germany; d Universidad de Sevilla, Sevilla, Spain.

**Keywords:** advanced fibrosis, nonalcoholic steatohepatitis, progression

## Abstract

One fifth of patients with nonalcoholic fatty liver disease (NAFLD) may progress to nonalcoholic steatohepatitis (NASH), which can increase the risk of cirrhosis, cancer, and death. To date, reported predictors of NASH progression have been heterogeneous.

We identified determinants of fibrosis progression in patients with NASH in the United States using physician-reported data from the real-world Global Assessment of the Impact of NASH (GAIN) study, including demographics and clinical characteristics, NASH diagnostic information, fibrosis stage, comorbidities, and treatment. We developed a logistic regression model to assess the likelihood of fibrosis progression since diagnosis, controlling for sociodemographic and clinical variables. An iterative nested model selection approach using likelihood ratio test determined the final model.

A total of 989 patients from the GAIN US cohort were included; 46% were women, 58% had biopsy-proven NAFLD, and 74% had fibrosis stage F0–F2 at diagnosis. The final multivariable model included age, years since diagnosis, sex, employment status, smoking status, obesity, fibrosis stage, diagnostic biopsy, Vitamin E, and liver transplant proposed at diagnosis. Odds of progression were 17% higher (odds ratio, 1.17 [95% CI: 1.11–1.23]; *P* < .001) with each year since NASH diagnosis, 41% lower (0.59 [0.38–0.90]; *P* = .016) for women than men, 131% higher (2.31 [1.30–4.03]; *P* = .004) for smokers versus non-smokers, and 89% higher (1.89 [1.26–2.86]; *P* = .002) with obesity. Odds of progression were also higher with part-time, retired, unemployed, and unable to work due to NASH status versus full-time employment, and when a liver transplant was proposed at diagnosis.

Disease duration and severity, obesity, smoking, and lack of full-time employment were significant determinants of fibrosis progression. These findings can support clinical and health-policy decisions to improve NASH management in the US.

## Abstract 2

Supplemental Digital Content is available in the text

## 1 Introduction

Nonalcoholic fatty liver disease (NAFLD) is prevalent in approximately one third of adults in the United States,^[1]^ with a rising prevalence driven by increasing rates of obesity and type 2 diabetes mellitus, which are particularly high among patients with NAFLD.^[2–4]^ The incidence of NAFLD in children and adolescents has grown to nearly 10% overall and 70% among those with obesity.^[5,6]^ Progression of NAFLD to nonalcoholic steatohepatitis (NASH) is characterized by steatosis, ballooning, and inflammation, with or without fibrosis, which can lead to cirrhosis and increase the risk of hepatocellular carcinoma and death.^[2,7]^ NASH is estimated to occur in 20% or more of patients with NAFLD, 3% to 4% of all Americans, and is more prevalent among Hispanic than Caucasian patients (19% vs 10%).^[8]^ Although the progressive nature of NASH has been shown to substantially impact health-related quality of life and mortality,^[7,9,10]^ there is no pharmacologic treatment approved specifically for NASH. Current management strategies include lifestyle interventions, primarily regular physical activity and nutrition to improve insulin sensitivity and lipid profile, antidiabetic and cardiometabolic medications, and Vitamin E, with the possibility of eventual bariatric surgery and/or liver transplantation in the setting of comorbid obesity or decompensated cirrhosis, respectively.^[11–13]^

The high prevalence and increasing incidence of NASH among both children and adults indicate a burgeoning population health concern with clinical, humanistic, and societal implications. The substantial burden of NASH makes appropriate identification and accurate clinical characterization of disease status and progression vitally important to clinical management decisions. The risks and limitations of confirmation with liver biopsy elevate the relevance of other factors that drive fibrosis progression in order to optimize clinical decision support strategies across a broad range of care settings. As such, more practical clinical indicators of NASH progression would be of value.

Further complicating the characterization and management of NASH is a notable inter-patient variability in disease progression and outcomes.^[14]^ The nature of this variability is multifaceted and complex, including factors related to genetics, lifestyle, the environment, and epigenetics, and our understanding of these considerations remains incomplete.^[15]^ Demographic factors such as age, sex, race, and ethnicity,^[16–19]^ as well as laboratory markers including *PNPLA3* gene polymorphisms, alanine aminotransferase, aspartate aminotransferase, and cholesterol measures have been associated with fibrosis score and/or NASH progression, but evidence of their roles remains inconsistent.^[16,18–21]^ Comorbid conditions and related measures of disease activity have also been associated with fibrosis worsening, including insulin resistance and hemoglobin A1c, body mass index and waist circumference, and extensive non-alcoholic fatty pancreas disease (NAF-P).^[16–19,21,22]^

The variability of evidence has hindered the application of more accessible clinical indicators of disease status. Predictors of NASH progression identified in health services research have not been consistent, and are often derived from small sample sizes in single countries or regions.^[23]^ In order to further inform clinical and public health decisions, we sought to identify meaningful sociodemographic and clinical determinants of fibrosis progression in patients with NASH in the US.

## 2 Methods

### 2.1 The GAIN study

**Figure 1:**
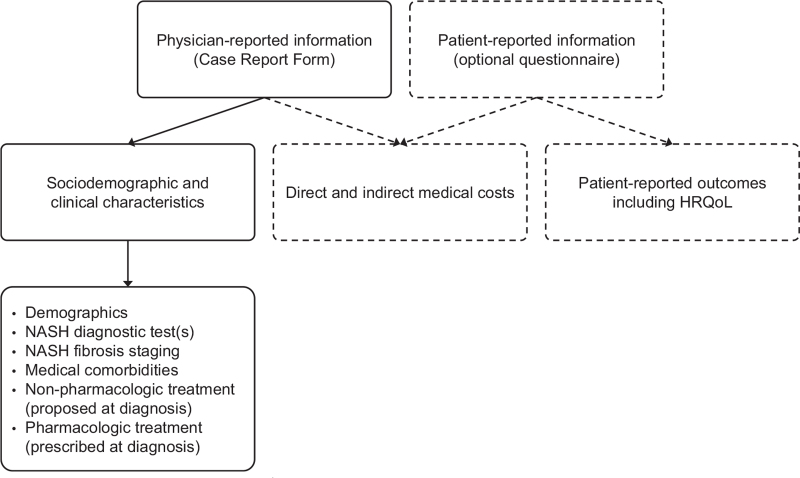
Participant outcomes used from the GAIN study^∗^ (adapted from O’Hara 2020^[9]^). GAIN = Global Assessment of the Impact of NASH, HRQoL = health-related quality of life, NASH = nonalcoholic steatohepatitis. ^∗^Dashed elements from the GAIN study were not included in the development of this model.

We used physician-reported data from the Global Assessment of the Impact of NASH (GAIN) study, conducted between June and October 2018, the design and methods of which have been reported previously (Fig. 1).^[9]^ Briefly, the Global Assessment of the Impact of NASH study (GAIN study) was a multinational, retrospective, and cross-sectional study of adults (≥18 years) with NASH that investigated patient characteristics, clinical and patient-reported outcomes, and NASH-related direct and indirect costs in the US and Europe (France, Italy, Germany, Spain and the United Kingdom). Eligible patients had confirmed NASH diagnosis at least 12 months prior to study recruitment. In order to approximate a real-world patient population, an eligible NASH diagnosis was defined as histologically confirmed NASH with fibrosis; biomarker evidence of advanced fibrosis in patients with metabolic syndrome risk factors (aspartate aminotransferase/alanine aminotransferase ratio, NAFLD fibrosis score, body mass index, aspartate aminotransferase/alanine aminotransferase ratio, diabetes, and Fibrosis-4 score); or imaging evidence of advanced fibrosis and/or cirrhosis in patients with metabolic syndrome risk factors (by ultrasound, magnetic resonance imaging, or computed tomography). All patients were classified as F0-F4 by physicians, including patients with a biopsy but also those diagnosed by biomarker or imaging. Advanced fibrosis was defined as F3 or F4. Participating physicians recruited eligible patients consecutively regardless of the reason for the visit, and provided demographic, clinical, and resource use information from the medical charts. Specialists such as hepatologists, gastroenterologists, endocrinologists, and diabetologists were recruited from clinics and hospitals. Patients could volunteer to complete questionnaires regarding health-related quality of life and non-medical/indirect costs, but this information was not used for this study. All patient participants provided written informed consent, which included consent for their data to be used in publications. The study protocol was approved by the Research Ethics Subcommittee of the Faculty of Health and Social Care within the University of Chester, and carried out in accordance with relevant guidelines including the Declaration of Helsinki.

### 2.2 Disease and outcome definitions

This study used the physician-reported patient demographic and clinical characteristics from the GAIN study, as well as NASH diagnostic procedure (biopsy, laboratory/biochemical markers, or imaging), time since diagnosis, fibrosis stage at diagnosis, medical comorbidities, non-pharmacological treatments proposed at NASH diagnosis, weight loss in the past 12 months, and pharmacological treatment prescribed at NASH diagnosis and still being taken by the patient. Demographic and clinical characteristics included age, sex, race/ethnicity, height, body mass index (numerical and categorical), alcohol consumption, smoking, and employment status. Fibrosis stage was reported by physicians at NASH diagnosis and at the date of consultation related to the study recruitment visit. Change in fibrosis stage determined whether a patient had progressed, regressed, or remained stable. Patients were stratified by time since NASH diagnosis. Medical comorbidity information included number of comorbid conditions and presence of obesity, diabetes, any cardiovascular condition, hypertension, and/or dyslipidemia. Non-pharmacological treatments proposed at diagnosis were classified as lifestyle interventions, diet, behavioral, bariatric surgery, and/or liver transplantation. Physician proposal of these interventions did not mean the patient received the intervention(s) proposed.

### 2.3 Model development and statistical analysis

Descriptive statistics were used to summarize patient demographic and clinical characteristics. Categorical variables were summarized as frequencies and percentages. Continuous variables were summarized as mean with standard deviation, or standard error and range, for normally distributed variables, and as mean and standard deviation as well as median, interquartile range, and range for non-normally distributed variables. No imputation of missing responses was conducted. Patients with missing responses for fibrosis progression (fibrosis stage recorded at 2 different time points) were excluded from the analysis. A multicollinearity analysis was conducted to assess interrelationships between variables and remove redundant variables. Scatter plots and Pearson moment correlation explored interrelationships between continuous covariates. Variables with higher correlations (*r* > .8) were removed to avoid bias in parameter estimation.

We developed a logistic regression model to assess the likelihood of fibrosis progression since diagnosis (as a binary outcome) while controlling for sociodemographic and clinical variables. Patients with stage F4 decompensated cirrhosis at baseline were excluded from the analysis because fibrosis progression was not possible beyond this stage. Details regarding the model covariates are included in Table S1 in the Supporting Appendix, http://links.lww.com/MD2/A748 (see Table, Supplemental Content, which outlines covariates considered for the regression model). Outliers were identified by assessment of influential observations using Cook's D (cut-off value of 4/sample size), leverage, and residuals. The normality assumptions were assessed via linearity assumption of the predictors and distribution of residuals. Variables with a significant association (*P* *<* .05) or trend (*P* > .05 and *P* *<* .10) in the univariable analysis were included in the multivariable model. An iterative nested model selection approach using the likelihood ratio test was conducted to determine the most appropriate final model based on complexity and goodness of fit. All analyses were performed using R software (The R Project; www.r-project.org).

## 3 Results

### 3.1 Patient characteristics and disease progression

A total of 1016 patients from the US cohort of the GAIN study had fibrosis stage reported for at least 2 time points and were included in this study. Twenty seven patients had stage F4 decompensated cirrhosis at baseline and were excluded (according to the study protocol, that there was no chance of further stage progression), leaving 989 patients in the analysis cohort. Nearly half of all patients were women (46%), and the mean age was 51 years (Table 1). Slightly more than half of patients underwent liver biopsy (58%) and the majority indicated fibrosis stage F0–F2 at diagnosis (74%; Table 1). Half of patients had either lifestyle modification and/or diet change proposed at diagnosis (50% each), few were prescribed lipid-lowering drugs (16%) or Vitamin E (14%) at diagnosis, and fewer had bariatric surgery (8%) or liver transplantation proposed at the time of diagnosis (3%; Table 1). One hundred forty one patients (14%) had evidence of fibrosis progression; 78 (8%) regressed and 770 (78%) were reported with stable disease stage (Table 2). When stratified by years since diagnosis, a lower proportion of patients with ≤5 years versus >5 years since diagnosis had progressed (12% and 42%, respectively).

**Table 1:** Baseline sociodemographic and clinical characteristics of the United States fibrosis gain study cohort.

Variable	No progression (n = 848)	Progressed (n = 141)	All patients (n = 989)
Age, mean (SD), yr	50.8 (10.9)	52.7 (10.3)	51.1 (10.8)
Sex, female, n (%)	407 (48)	47 (33)	454 (46)
Race or ethnicity, n (%)			
White	28 (3)	3 (2)	31 (3)
Other	820 (97)	138 (98)	958 (97)
Height, mean (SD), meters	1.7 (0.2)	1.7 (0.2)	1.7 (0.2)
BMI, mean (SD), kg/m^2^	32.8 (13.5)	33.6 (12.3)	32.9 (13.4)
BMI, physician-reported			
Underweight	10 (1)	3 (2)	13 (1)
Normal	186 (22)	27 (19)	213 (22)
Overweight	156 (18)	26 (18)	182 (18)
Obese	496 (59)	85 (60)	581 (59)
Alcohol consumption, n (%)			
Never	369 (44)	40 (28)	409 (41)
Once per mo	248 (29)	42 (30)	290 (29)
2 to 4 times per mo	133 (16)	35 (25)	168 (17)
4 to 6 times per mo	50 (6)	17 (12)	67 (7)
More than 6 times per mo	11 (1)	3 (2)	14 (1)
Unknown	37 (4)	4 (3)	41 (4)
Smoking status, n (%)			
Non-smoker	457 (54)	60 (43)	517 (52)
Ex-smoker	256 (30)	44 (31)	300 (30)
Current smoker	93 (11)	28 (20)	121 (12)
Unknown	42 (5)	9 (6)	51 (5)
Employment, n (%)			
Full-time	430 (51)	45 (32)	475 (48)
Part-time	142 (17)	30 (21)	172 (17)
Self-employed	79 (9)	12 (9)	91 (9)
Student	16 (2)	3 (2)	19 (2)
Retired	72 (9)	23 (16)	95 (10)
Unemployed	62 (7)	16 (11)	78 (8)
Homemaker	36 (4)	8 (6)	44 (4)
Physically unable to work due to NASH or related complications	3 (0.4)	3 (2)	6 (1)
Physically unable to work due to other reason(s)	8 (1)	1 (1)	9 (1)
Years since diagnosis			
Mean (SD)	2.1 (2.4)	4.4 (5.1)	2.4 (3.0)
Median (IQR)	1.2 (0.9, 27.9)	2.1 (1.0, 21.0)	1.3 (0.9, 27.9)
Liver biopsy, n (%)	488 (58)	90 (64)	578 (58)
Fibrosis stage at diagnosis, n (%)			
F0	171 (20)	30 (21)	201 (20)
F1	240 (28)	53 (38)	293 (30)
F2	192 (23)	41 (29)	233 (24)
F3	121 (14)	15 (11)	136 (14)
F4CC	124 (15)	2 (1)	126 (13)
Number of comorbidities, n (%)			
0	310 (37)	32 (23)	342 (35)
1	207 (24)	30 (21)	237 (24)
2	141 (17)	19 (14)	160 (16)
≥3	190 (22)	60 (43)	250 (25)
Comorbidities, n (%)			
Obesity from calculated BMI	496 (59)	85 (60)	581 (59)
Obesity from physician notes	332 (39)	83 (59)	415 (42)
Type 2 diabetes mellitus	203 (24)	40 (28)	243 (25)
Cardiovascular disease	22 (3)	8 (6)	30 (3)
Hypertension	234 (28)	53 (38)	287 (29)
Dyslipidemia	257 (30)	56 (40)	313 (32)
Weight loss, past 12 mo			
0	820 (97)	133 (94)	953 (97)
<5%	10 (1)	3 (2)	13 (1)
5% to <7%	8 (1)	3 (2)	11 (1)
7% to 10%	6 (1)	1 (1)	7 (1)
>10%	3 (0.4)	1 (1)	4 (0.4)
Missing data	1	0	1
Non-pharmacological NASH treatment proposed at diagnosis			
Lifestyle change	415 (49)	83 (59)	498 (50)
Diet change	409 (48)	81 (57)	490 (50)
Behavioral strategies	28 (3)	3 (2)	31 (3)
Bariatric or intragastric surgery	66 (8)	16 (11)	82 (8)
Liver transplantation	19 (2)	10 (7)	29 (3)
Pharmacological NASH treatment			
Lipid-lowering drugs	126 (15)	28 (20)	154 (16)
Vitamin E	128 (15)	12 (9)	140 (14)
Metformin	99 (12)	20 (14)	119 (12)
Sulfonylurea	2 (0.2)	0	2 (0.2)
Thiazolidinediones	23 (3)	14 (10)	37 (4)
GLP-1 receptor agonist	8 (1)	0	8 (1)
SGLT-2	1 (0.1)	0	1 (0.1)
DPP-4	1 (0.1)	0	1 (0.1)
Other anti-diabetic medication(s)	18 (2)	3 (2)	21 (2)
Other medication(s)	60 (7)	16 (11)	76 (8)

BMI = body mass index, CC = compensated cirrhosis, DPP-4 = dipeptidyl peptidase-4, GAIN = Global Assessment of the Impact of NASH study, GLP-1 = glucagon-like peptide-1, NASH = nonalcoholic steatohepatitis, SD = standard deviation, SGLT-2 = sodium-glucose co-transporter-2. Proportions may not sum to 100% due to rounding.

**Table 2:** Disease progression status stratified by years since non-alcoholic steatohepatitis diagnosis.

Variable, n (%)	Progressed	Regressed	Stable
All patients (n = 989)	141 (14)	78 (8)	770 (78)
Patients with ≤5 yr since diagnosis (n = 898)	103 (12)	74 (8)	721 (80)
Patients with >5 yr since diagnosis (n = 91)	38 (42)	4 (4)	49 (54)

### 3.2 Univariable analysis

Based on the results of the multicollinearity analysis, the univariable model excluded highly correlated covariates. Exploratory assessment of the model covariates by univariable regression, where each variable was evaluated independently without consideration of other covariates, suggested several potentially influential factors for fibrosis progression (Table 3). Significant associations with fibrosis progression were observed for years since NASH diagnosis, female sex, greater alcohol consumption, less than full-time employment status, being a current smoker, and stage F4 fibrosis (compensated cirrhosis) at diagnosis. Significant comorbidities included obesity (by physician assessment) and dyslipidemia. Factors related to proposed lifestyle modification and prescribed pharmacological treatments (and still received at last consultation date) such as Vitamin E, thiazolidinediones, or other anti-diabetic medications at diagnosis were also significant, as was history of liver transplant proposed at diagnosis.

**Table 3:** Modeling determinants of fibrosis progression.

Independent variable	Univariable model	Multivariable model	Final model
	OR (95% CI), *P* value	OR (95% CI), *P* value	OR (95% CI), *P* value
Age (continuous)	1.02 (1.00–1.03), *P =* .050	1.01 (0.99–1.03), *P =* .457	1.01 (0.99–1.03), *P =* .310
Sex, female vs male	0.54 (0.37–0.78), *P =* .001	0.60 (0.39–0.92), *P =* .019	0.59 (0.38–0.90), *P =* .016
Race/ethnicity, white vs other	0.66 (0.16–1.90), *P =* .500	–	–
Alcohol consumption, vs never			–
Once per mo	1.57 (0.99–2.50), *P =* .056	1.23 (0.73–2.06), *P =* .441	
2 to 4 times per mo	2.43 (1.48–3.98), *P <* .001	1.54 (0.86–2.76), *P =* .142	
4 to 6 times per mo	3.14 (1.62–5.88), *P <* .001	1.49 (0.66–3.21), *P =* .317	
More than 6 times per mo	2.52 (0.55–8.46), *P =* .170	1.60 (0.29–6.65), *P =* .542	
Unknown	1.00 (0.29–2.65), *P =* .996	0.53 (0.13–1.68), *P =* .323	
Smoking status, vs non-smoker			
Ex-smoker	1.31 (0.86–1.99), *P =* 2.00	1.20 (0.74–1.96), *P =* .456	1.28 (0.79–2.04), *P =* .311
Current smoker	2.29 (1.38–3.76), *P =* .001	2.01 (1.09–3.66), *P =* .023	2.31 (1.30–4.03), *P =* .004
Unknown	1.63 (0.71–3.38), *P =* .212	2.31 (0.89–5.52), *P =* .069	1.92 (0.77–4.40), *P =* .138
Employment, vs full-time			
Part-time	2.03 (1.22–3.34), *P =* .005	1.80 (1.02–3.13), *P =* .039	1.75 (1.00–3.01), *P =* .046
Self-employed	1.45 (0.71–2.79), *P =* .283	0.91 (0.40–1.94), *P =* .821	0.92 (0.41–1.93), *P =* .830
Student	1.79 (0.41–5.64), *P =* .368	1.93 (0.39–7.08), *P =* .360	1.83 (0.38–6.59), *P =* .397
Retired	3.05 (1.72–5.31), *P <* .001	2.22 (1.07–4.51), *P =* .030	2.06 (1.01–4.14), *P =* .045
Unemployed	2.47 (1.28–4.55), *P =* .005	2.32 (1.12–4.66), *P =* .020	2.08 (1.02–4.11), *P =* .039
Homemaker	2.12 (0.87–4.64), *P =* .074	1.96 (0.73–4.79), *P =* .157	2.07 (0.78–5.00), *P =* .120
Physically unable to work due to NASH or related complications	9.56 (1.73–52.97), *P =* .007	28.76 (3.37–273.34), *P =* .002	26.63 (3.44–238.94), *P =* .002^∗^
Physically unable to work due to other reason(s)	1.19 (0.06–6.72), *P =* .868	0.71 (0.03–6.52), *P =* .799	0.54 (0.02–4.46), *P =* .629
Years since diagnosis (continuous)	1.18 (1.13–1.24), *P <* .001	1.15 (1.09–1.21), *P <* .001	1.17 (1.11–1.23), *P <* .001
Liver biopsy, yes vs no	1.30 (0.91–1.90), *P =* .159	1.52 (1.00–2.33), *P =* .053	1.49 (0.98–2.28), *P =* .062
Fibrosis stage at diagnosis, vs F0			
F1	1.26 (0.78–2.07), *P =* .356	0.99 (0.58–1.71), *P =* .962	1.00 (0.58–1.72), *P =* .987
F2	1.22 (0.73–2.05), *P =* .454	0.85 (0.47–1.54), *P =* .592	0.87 (0.49–1.57), *P =* .647
F3	0.71 (0.36–1.35), *P =* .304	0.39 (0.17–0.82), *P =* .016	0.38 (0.17–0.80), *P =* .014
F4CC	0.09 (0.01–0.31), *P =* .001	0.06 (0.01–0.23), *P =* .001	0.06 (0.01–0.23), *P <* .001
Comorbidities			
Obesity from calculated BMI, yes vs no	1.07 (0.75–1.55), *P =* .700	–	–
Obesity from physician notes, yes vs no	2.22 (1.55–3.20), *P <* .001	1.78 (1.15–2.76), *P =* .010	1.89 (1.26–2.86), *P =* .002
Type 2 diabetes mellitus, yes vs no	1.26 (0.84–1.86), *P =* .262	–	–
Cardiovascular disease, yes vs no	2.26 (0.93–4.98), *P =* .055	–	–
Hypertension, yes vs no	1.58 (1.08–2.28), *P =* .016	0.99 (0.60–1.62), *P =* .973	–
Dyslipidemia, yes vs no	1.51 (1.04–2.18), *P =* .027	0.95 (0.57–1.58), *P =* .853	–
Non-pharmacological NASH treatment proposed at diagnosis			
Lifestyle change, yes vs no	1.49 (1.04–2.15), *P =* .031	1.12 (0.71–1.76), *P =* .619	–
Behavioral strategies, yes vs no	0.64 (0.15–1.83), *P =* .461	–	–
Bariatric or intragastric surgery, yes vs no	1.51 (0.82–2.64), *P =* .159	–	–
Liver transplantation, yes vs no	3.33 (1.46–7.17), *P =* .003	3.96 (1.35–11.40), *P =* .011	4.95 (1.77–13.55), *P =* .002
Pharmacological NASH treatment^†^			
Lipid-lowering drugs, yes vs no	1.42 (0.89–2.21), *P =* .133	–	–
Vitamin E, yes vs no	0.52 (0.27–0.94), *P =* .040	0.52 (0.25–1.01), *P =* .063	0.54 (0.27–1.02), *P =* .070
Metformin, yes vs no	1.25 (0.73–2.06), *P =* .400	–	–
Thiazolidinediones, yes vs no	3.95 (1.94–7.79), *P <* .001	2.03 (0.78–5.06), *P =* .135	–
Other anti-diabetic medication(s), yes vs no	1.00 (0.23–3.01), *P =* .998	–	–
Other medication(s), yes vs no	1.68 (0.91–2.94), *P =* .081	–	–

BMI = body mass index, CC = compensated cirrhosis, CI = confidence interval, NASH = nonalcoholic steatohepatitis, OR = odds ratio.

∗ Only 6 patients reported inability to work due to NASH; this odds ratio should be interpreted with caution.

† Pharmacological treatment prescribed at diagnosis and receiving it at date of last consultation. Weight loss in the past 12 months, expressed as a percentage, was not significant in the univariable model (All *P* > .05).

### 3.3 Multivariable model determination and final model selection

The multivariable logistic regression model revealed several significant determinants of fibrosis progression, including age, years since diagnosis, sex, employment status, smoking status, obesity, fibrosis stage, comorbidities, and treatment (Table 3). Covariates remained in the final model if an individual predictor or a component of a predictor (e.g., an employment category) was significant in the multivariable model, or if the predictor was known to be a determinant of progression based on published evidence. The predictors of progression that remained in the final multivariable model included years since diagnosis, sex, employment status, smoking status, obesity, fibrosis stage, diagnostic biopsy, Vitamin E, and liver transplant proposed at diagnosis (Table 3). Based on the final model, the odds of progression may be expected to be 17% higher with each year since NASH diagnosis when all other covariates are held constant. The odds of progression were 41% lower for women than men, 131% higher for current smokers versus non-smokers, 89% higher for those with obesity recorded by their physician, and 4 times higher for those where a liver transplant was proposed at the time of NASH diagnosis. Compared with patients with full-time employment, the odds of progression were substantially higher for those with part-time employment (+75%) and patients who were retired (+106%), unemployed (+108%), or physically unable to work due to NASH or related complications (of note, only 6 patients [0.6%] were unable to work due to NASH diagnosis).

## 4 Discussion

This study identified sociodemographic and clinical factors associated with disease progression in adults with NASH in the United States. Significant predictors of progression were related to disease duration and severity, sex, employment, smoking, obesity, diagnosis by liver biopsy and NASH treatment. Longer disease duration and greater severity along with presence of obesity, smoking, and lack of full-time employment were each particularly influential factors for fibrosis progression in the final model. Specifically, this study predicted the odds of progression to be 41% lower for women than men, 131% higher for smokers, 89% higher for those with obesity, and 395% higher for those whose physician recommended liver transplantation at the time of diagnosis. The odds of progression were lower for patients with full-time employment compared with those who were employed part-time, retired, or unemployed, when accounting for all other factors.

Based on our model, the odds of progression may be expected to increase 17% with each year since NASH diagnosis, suggesting progression to a higher stage every 5.9 years on average. This finding is generally aligned with the 7.1 years reported by Singh and colleagues (2015) in a large meta-analysis of studies comparing paired liver biopsies between patients with NAFLD or NASH.^[24]^ Other predictive modeling studies of NASH and advanced fibrosis have tended to focus on factors associated with the presence of disease rather than changes in disease, such as progression. Our findings were generally consistent with other modeling studies in US patients, though we were able to utilize a large sample of real-world physician-reported information from medical charts to identify predictors of disease progression. Rosenblatt and colleagues (2019) reported from a sample of 91 patients that the presence of NAF-P was predictive of advanced fibrosis but not NASH (extensive NAF-P was predictive of both advanced fibrosis and NASH).^[22]^ We did not investigate NAF-P as a predictor of disease progression due to lack of data on this condition in our sample, though from a clinical perspective NAF-P may be considered more suited to predicting disease rather than disease severity. Bazick and colleagues (2015) investigated predictors of advanced fibrosis or NASH in 100 US patients with type 2 diabetes, reporting white race, body mass index, waist circumference, and certain metabolic biomarkers (e.g., alanine aminotransferase, aspartate aminotransferase, hemoglobin A1c) to be predictive of NASH.^[16]^ Age, Hispanic ethnicity, body mass index, waist-to-hip ratio, hypertension, and other biomarkers (e.g., alanine aminotransferase-to-aspartate aminotransferase ratio) were predictive of advanced fibrosis. We analyzed a broader population of patients with NASH, but did not have the same breadth of biomarker results available as Bazick and colleagues, but we did capture clinical parameters that are easily accessible in the patient's medical history in a broader NASH population.

These findings should be interpreted in the context of certain strengths and limitations. This study utilized a large sample of patients from the GAIN study, with physician-reported information derived from patients’ medical charts. The nature of the dataset and subsequent findings should provide a pragmatic, useful basis for clinical decision-making, which was our objective. A possible limitation was the inclusion of misdiagnosed (i.e., incorrect distinction between NAFL and NASH) and misclassified NASH cases because of the use of noninvasive testing to diagnose disease. However, including only patients in the GAIN study with biopsy-confirmed disease would have focussed the data on a potentially unrepresentative cohort of patients in secondary and/or tertiary care, which would have missed the complexity and diversity of this condition.^[9]^ The reporting of comorbidities from a single physician's notes may have led to underreporting for this variable, but the observed frequency of comorbidities appeared consistent with the known epidemiology and risk factors of NAFLD. Patients from the GAIN US cohort had a mean of 2.4 years since diagnosis, though this was higher among those who had evidence of disease progression versus those who did not (4.4 and 2.1 years, respectively). The cohort included patients with and without liver biopsy, which was associated with progression and may have influenced the characterization of fibrosis stage (also a significant predictor of progression), as biopsy is the most sensitive diagnostic procedure (the gold standard for confirmation of the NASH diagnosis and fibrosis status), and patients with liver biopsy may have been suspected to have had more advanced disease.^[11]^ Orders for a liver biopsy are likely indicative of advanced disease based on the physician's clinical assessment. Frequency of alcohol consumption is ultimately a patient-reported outcome with potential for reporting bias and is a proxy for total alcohol consumption, but was not significant in the final model. We also used more than 1 definition of obesity, with the physician-reported assessment of obesity remaining in the final model, which may have influenced the results. Pharmacological treatment prescribed at diagnosis and still reported as being taken by the patient did not objectively confirm actual administration of medications. Similarly, non-pharmacological treatments were proposed at diagnosis, and we were not able to identify which interventions were put into effect by the patients, nor to what extent. Weight loss in the last 12 months was not deemed to be significant in the final model for reasons the authors attribute to the data collection. Weight loss was captured as a percentage of reduced weight by physician recall over 12 months, rather than more specific measurements and time points, which was determined inappropriate for interpretation and inclusion in the model. The employment category of patients reporting inability to work specifically due to NASH was included in the final model but comprised a very small sample (6 patients) and the odds ratio should be interpreted with caution.

In summary, we reported significant predictors of fibrosis progression in US adults with NASH that may be applied in the clinic to support disease status evaluation and management. We used physician-reported, medical chart-derived clinical and socioeconomic information from a large sample of adults with NASH in the US, suggesting that disease duration and severity, sex, employment, smoking, obesity, treatment practices, and diagnosis with liver biopsy predict disease progression in patients with this chronic condition. NAFLD is a complex, dynamic, and heterogeneous condition that requires a multidisciplinary approach and consideration of socioeconomic factors. These findings may support point-of-care and public health decisions and should be further validated in large real-world patient populations.

## Author contributions

**Conceptualization:** Leonardo Ruiz-Casas, Gabriel Pedra, Anum Shaikh, Bethany Franks, Harpal Dhillon.

**Formal analysis:** Leonardo Ruiz-Casas, Gabriel Pedra, Anum Shaikh, Bethany Franks, Harpal Dhillon, João Diogo da Rocha Fernandes, Kamal Kant Mangla, Margarida Augusto, Jörn M. Schattenberg, Manuel Romero-Gómez.

**Writing – review & editing:** Leonardo Ruiz-Casas, Gabriel Pedra, Anum Shaikh, Bethany Franks, Harpal Dhillon, João Diogo da Rocha Fernandes, Kamal Kant Mangla, Margarida Augusto, Jörn M. Schattenberg, Manuel Romero-Gómez.
